# Unveiling Morphine: A Rapid and Selective Fluorescence Sensor for Forensic and Medical Analysis

**DOI:** 10.3390/s24061722

**Published:** 2024-03-07

**Authors:** Ramin Boroujerdi, Andrew Butt, Richard Paul, Santanu Majumder

**Affiliations:** Faculty of Science and Technology, Bournemouth University, Talbot Campus, Fern Barrow, Poole BH12 5BB, UK; aabutt@bournemouth.ac.uk (A.B.); rpaul@bournemouth.ac.uk (R.P.); smajumder@bournemouth.ac.uk (S.M.)

**Keywords:** morphine, toxicology, turn-off sensor, forensic biological fluids, CCD-RSM

## Abstract

Opioid use, particularly morphine, is linked to CNS-related disorders, comorbidities, and premature death. Morphine, a widely abused opioid, poses a significant global health threat and serves as a key metabolite in various opioids. Here, we present a turn-off fluorescent sensor capable of detecting morphine with exceptional sensitivity and speed in various samples. The fluorescent sensor was developed through the dimerization process of 7-methoxy-1-tetralone and subsequent demethylation to produce the final product. Despite morphine possessing inherent fluorophoric properties and emitting light in an approximately similar wavelength as the sensor’s fluorescent blue light, the introduction of the target molecule (morphine) in the presence of the sensor caused a reduction in the sensor’s fluorescence intensity, which is attributable to the formation of the sensor–morphine complex. By utilizing this fluorescence quenching sensor, the chemo-selective detection of morphine becomes highly feasible, encompassing a linear range from 0.008 to 40 ppm with an impressive limit of detection of 8 ppb. Consequently, this molecular probe demonstrates a successful application in determining trace amounts of morphine within urine, yielding satisfactory analytical results. The study also explores the effect of several variables on the sensor’s response and optimizes the detection of morphine in urine using a response surface methodology with a central composite design.

## 1. Introduction

The illegal usage of controlled substances and illicit drugs presents a significant threat to human well-being, family cohesion, and societal equilibrium [1,2,3]. Often, there are indications of the use or abuse of such drugs found at the crime scene [4,5,6,7]. This has made the rapid and accurate detection of such drugs a compelling subject of research. Moreover, many of these chemical compounds hold immense value in the medical field due to their significance in patient recovery and overall health [8,9,10]. Consequently, pharmaceutical industries stand to gain considerable benefits from the availability of sensitive and selective sensors for quality control, ensuring the production of pure products and the monitoring of safe dosages in modern drug-delivery setups [11,12,13,14,15]. In medicine, an early diagnosis of patients’ conditions (particularly in cases of overdose resulting from drug abuse, misuse, or drug–drug interactions) can be life-saving [16,17,18,19,20,21]. Consequently, scientists are actively working on the development of novel sensors that offer better sensitivity, selectivity, ease of use, fast response, and affordability for the on-site and in-lab detection of illicit drugs [22,23,24]. Such advancements also hold tremendous importance in the prevention and reduction of drug-related crimes.

Morphine, one of the most powerful analgesics in use worldwide [25,26], is primarily extracted from the opium poppy plants [27]. It is a commonly used narcotic drug with extensive applications in clinical medicine [28,29,30]. One of the most significant drawbacks of morphine is its high potential for addiction, making it prone to abuse [31,32,33]. Morphine induces a rewarding effect and hyperlocomotion, which lead to addiction through a dopamine-reward pathway [34,35]. Morphine targets and binds to the μ-opioid receptor in the brain, and this binding leads to an inhibition of the release of a range of neurotransmitters, such as GABA [36]. This inhibition leads to an increase in dopamine release in the brain’s reward system, particularly in the nucleus accumbens [37,38]. The surge of dopamine creates a feeling of pleasure and euphoria, reinforcing the rewarding effects of morphine and contributing to its potential for abuse [39,40]. Additionally, this interaction with the dopamine-reward pathway is responsible for the pain-relieving properties of morphine, as it alters the perception of pain signals in the brain [41,42]. Intentionally administered morphine or morphine generated through the metabolism of other drugs, such as heroin, may potentially cause respiratory depression and even lead to fatal outcomes when taken in high doses [43,44].

A variety of methods have been developed for the detection of controlled and illicit drugs; they mainly include high-performance liquid chromatography (HPLC) [45,46,47], gas chromatography mass spectrometry (GCMS) [48,49,50], electrochemical sensors [7,51,52], electrochemical luminescence [53,54,55], fluorescence spectroscopy [56,57], and surface-enhanced Raman spectroscopy (SERS) [58,59,60]. However, these methods have certain shortcomings, such as a complexity in analysis, the need for highly trained operators, long pre-treatment periods, and high costs [59,61,62]. Fluorescence spectroscopy, with the aid of chemical fluorophores or biochemical aptamers, stands out as a promising detection technique that can overcome the limitations of the mentioned methods. The key strengths of this technique, other than low cost, simple preparation, and ease of use, lie in its high sensitivity and rapid response, and its ability to eliminate a wide range of interferences without requiring any pretreatment, as many compounds do not generate fluorescence emissions [1,63,64,65]. Moreover, fluorescence sensors have the potential to be fine-tuned by anchoring selective functional groups to enhance their selectivity for compounds like morphine and other opioids in various solutions [56,57].

Other than the importance of rapid detection of overdose and poisoning cases in medicine, the early hours of a crime scene investigation are extremely important for forensic analysts, as they play a critical role in obtaining vital information and insights, as well as acquiring data [66,67,68]. Portable and handheld spectrofluorometers, integrated with selective and highly sensitive chemical sensors, can be employed to facilitate quantitative and highly accurate presumptive tests [69,70,71]. Such selective chemical sensors also hold the promise of being utilized as confirmatory detection methodologies using cutting-edge stationary spectrofluorometers within laboratory environments as well. This paper introduces a novel selective fluorophore enabling the rapid, selective, and sensitive detection of morphine in various samples, with a particular focus on urine, a critical pharmaceutical and forensic biological fluid. The study employs Central Composite Design-Response Surface Methodology (CCD-RSM) as a systematic approach to analyze the influence of various variables on the sensor’s response to morphine in urine samples while optimizing the sensor’s performance to its fullest potential.

## 2. Experimental Section

### 2.1. Materials

All chemical reagents and solvents were purchased from Fisher Scientific (Loughborough, UK) and were of analytical reagent grade. Morphine, benzylpiperazine, nicotine, midazolam, and ketamine were purchased from either Sigma–Aldrich (Gillingham, UK) or LGC Standards (Middlesex, UK). Urine samples were voluntarily provided by the authors of this study (Ethics ID: 55013).

### 2.2. Software

The experimental design analysis and subsequent regression analysis of the urine analysis data were conducted using Design-Expert, statistical software version 11.0 (Stat-Ease, Minneapolis, MN, USA). The creation of figures and mathematical correction of baselines (when necessary and as indicated in the main text) were conducted using OriginPro 2022 (OriginLab, Northampton, MA, USA). Fluoracle (Edinburgh Instruments, Livingston, UK) was used as the operating software for the spectrofluorometer.

### 2.3. Instrumentation

Photoluminescence (PL) spectra were acquired using an FS5 Spectrofluorometer (Edinburgh Instruments, UK) equipped with a PMT-EXT detector, providing spectral coverage up to 980 nm. A quartz (170–2700 nm) micro-fluorescence cuvette with the designated volume of 0.7 mL and minimal volume of 0.1 mL was used for PL studies. Fourier-transform infrared (FTIR) spectra were recorded using an ATR-FTIR spectrophotometer (Agilent, Santa Clara, CA, USA). Raman spectra were collected with an XploRA™ PLUS Raman spectrometer (Horiba, Northampton, UK), equipped with a 532 nm laser and 50× lens.

### 2.4. Synthesizing the Sensor

Previously, 7,7′-Dimethoxy-1,1′-binaphthalene and its modified derivative, 7′-Methoxy-[1,1′-binaphthalen]-7-ol, were synthesized as part of a published research endeavor [72]. We have now embarked on a fresh scientific exploration, one that harnesses the potential of these synthesized materials in an entirely novel context. The optimized synthesis will briefly follow as outlined below:

The process of synthesis of 7,7′-Dimethoxy-1,1′-binaphthalene began with the mixing of 10 g of 7-methoxy-1-tetralone and 0.1 g of HgCl_2_ in a solution of 50 mL of toluene and 30 mL of ethanol. The addition of 1.6 g of aluminum foil resulted in a transformation of the solution to a grayish color. After 6 h of reflux heating, 50 mL of 10% diluted HCl were introduced in two portions, causing the solution to change to white and then yellow. Following the separation and drying of the organic phase, an orange solution was obtained. When subjected to cooling and agitation, yellow crystals were formed, which were subsequently filtered and washed with acetic anhydride and acetic acid.

**Figure 1:**
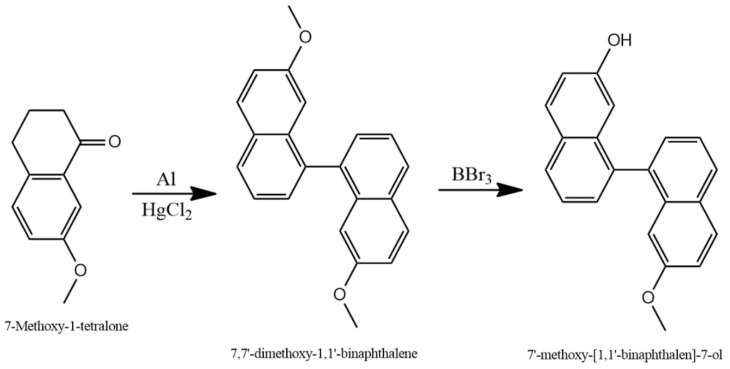
Synthesis process of 7,7′-Dimethoxy-1,1′-binaphthalene and 7′-Methoxy-[1,1′-binaphthalen]-7-ol.

The resulting compound was demethylated by dissolving 4 g of it in 15 mL of dichloromethane to produce 7′-Methoxy-[1,1′-binaphthalen]-7-ol. At −78 °C, 2 mL of BBr_3_ were added drop by drop, and the mixture was stirred for 3 h. Subsequently, 2 g of ice were introduced, leading to the conversion of one of the methoxy groups of the molecule into an alcohol group (Figure 1). After an additional hour, the organic phase was carefully separated, washed with water, and dried under a fume hood.

### 2.5. Standard Solutions

Owing to the inherent degradation of morphine within aqueous matrices, resulting primarily in the generation of pseudomorphine, alongside the less frequent emergence of morphine-N-oxide and potentially apomorphine [73], a deliberate choice has been made to formulate standard solutions within a methanolic medium. This selection is underpinned by the advantageous miscibility properties exhibited by water and methanol, ensuring the preservation of morphine’s integrity and stability, as well as facilitating its blending with water-based biological fluids, such as urine. Simultaneously, the developed sensing material demonstrates significantly enhanced solubility in methanol compared to water, owing to its distinct polarity characteristics.

The developed sensor, 7′-Methoxy-[1,1′-binaphthalen]-7-ol, was dissolved in methanol within a separate container and then introduced into the standard solutions. This infusion was carefully calibrated to achieve a final concentration of 100 ppm for the sensing material within the testing standards. Standard solutions of morphine were produced using pure methanol, encompassing a concentration gradient spanning from 1 ppb to 100 ppm. This comprehensive range was established to investigate the sensor’s linear response capabilities.

### 2.6. Biological Sample Preparation

Each urine sample was prepared by initially transferring 2 mL of untreated urine into a container. Subsequently, the requisite volumes of both the morphine solution (in methanol) and the sensor solution (in methanol) were added to the mixture (as illustrated in Table 1 and Table 2). To achieve a final sample volume of 5 mL, methanol was added into the solution. Adjustments to the concentrations of the sensor and morphine were made to align with the targeted concentrations dictated by the experimental design within the CCD-RSM framework.

### 2.7. Measurements of Photophysical Properties

Excitation and emission mapping were employed to determine the optimal excitation wavelength for the developed sensor solution (400 ppm in methanol). This mapping procedure involved measuring the fluorescence emission spectra within the range of 320 to 800 nm while systematically varying the excitation wavelength from 225 to 450 nm. The excitation wavelength associated with the maximal intensity of the fluorescence peak was ascertained as the optimal excitation wavelength. This wavelength was subsequently utilized to measure the fluorescence emission of the sensor in the presence of various drugs and in urine samples. Additionally, the same excitation wavelength was used to analyze the emission spectra of morphine in the absence of the sensor for comparison.

### 2.8. FTIR and Raman Spectroscopy

Regarding the FTIR and Raman spectroscopy, sample preparation necessitated the utilization of approximately 0.05 mg of dry, powdered samples. These samples were then transformed into tablets directly on the diamond surface of the ATR-FTIR instrument through the utilization of a specialized sample holder integrated with the ATR system. Subsequently, the compressed powder was delicately transferred onto a pristine glass sample holder for subsequent Raman spectroscopic analysis, subsequent to FTIR assessment.

For FTIR analysis, signal enhancement was accomplished through the accumulation of data derived from 24 scans of the identical sample, thereby augmenting the signal-to-noise ratio. Spectral data acquisition encompassed a range spanning from 650 to 3650 cm^−1^.

Concerning Raman spectroscopy, a laser beam having a wavelength of λ_ex_ = 532 nm was employed, accompanied by a grating featuring 600 grooves per millimeter, a 50% filter, a 100 μm slit width, and a 200 μm aperture. Data were accumulated over a period of 10 s, with a total accumulation count of 8, and a Read-to-Determine (RTD) time interval of 10 s. The acquisition span for Raman spectra encompassed the range from 0 to 3500 cm^−1^. This rigorous approach to sample preparation and data collection serves to ensure the veracity and precision of the spectroscopic outcomes.

### 2.9. CCD-RSM Design

The primary objective of this research is to create a chemo-selective probe capable of analyzing samples on site with minimal or no prior sample pretreatment. To address the challenge posed by the high miscibility of methanol and water, we utilized a solution containing the dissolved sensor (in methanol) and untreated urine to assess the sensor’s response. On-site urine drug-testing products provide a valuable opportunity for conducting immediate drug screenings directly at the point of care [74]. Due to the significance of on-site drug testing in the context of crime scene investigations, emergency rooms, and dependency clinics [75,76,77,78], it is crucial to understand how certain variables influence the sensor’s response. This understanding is essential for ensuring the sensor’s consistency to generate reliable results, underscoring the importance of comprehensive performance evaluations. These evaluations serve to highlight any limitations associated with the developed testing products.

The current investigation employed a simultaneous optimization technique known as Central Composite Design (CCD) within the framework of Response Surface Methodology (RSM). This integrated approach combines aspects of experimental design, mathematical modeling, and optimization, providing a systematic means to explore intricate response functions with a streamlined set of variable combinations [79,80,81,82]. In this research, we utilized a 24 full-factorial design in the CCD format to create mathematical models that would enable a quantitative assessment of sensor behavior under the influence of various parameters. We systematically explored the influence of alterations in morphine and sensor concentrations, temperature variations, and the duration since the mixing of morphine and the sensor on the fluorescence emission intensity response of the sensor. The experimental design employed a five-level CCD, resulting in a total of 30 experimental runs to facilitate the optimization process. Table 1 and Table 2 provide an overview of the five-level CCD used to explore the effects of the aforementioned variables on electrical current, highlighting its utility in optimizing sensor performance.

## 3. Results and Discussion

### 3.1. Fourier-Transform Infrared Spectroscopy

**Figure 2:**
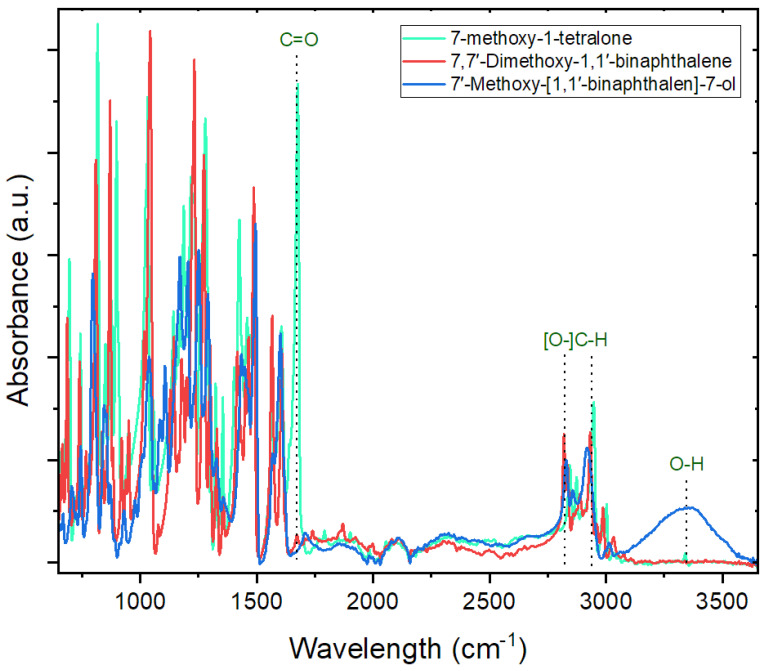
Comparison between the FTIR spectra of 7-methoxy-1-tetralone, 7,7′-Dimethoxy-1,1′-binaphthalene, and 7′-Methoxy-[1,1′-binaphthalen]-7-ol.

Figure 2 depicts the spectra of 7-methoxy-1-tetralone, its dimerized form 7,7′-Dimethoxy-1,1′-binaphthalene, and its derivative 7′-Methoxy-[1,1′-binaphthalen]-7-ol. In the spectrum of 7-methoxy-1-tetralone, a prominent C=O stretching bond is observed at approximately 1674 cm^−1^ [83]. This peak vanishes in both dimerized structures, confirming that dimerization occurred at the carbonyl site. A comparison of the spectra of 7,7′-Dimethoxy-1,1′-binaphthalene and 7′-Methoxy-[1,1′-binaphthalen]-7-ol reveals two new peaks: C−O stretching at 1106 cm^−1^ [84] and O−H stretching at 3349 cm^−1^ [85]. The presence of O-H peaks confirms the conversion of the functional group on the diene from methoxy to an alcoholic group.

It has been established that demethylation by a strong reagent such as BBr_3_ can either convert all [86] or just half [72,87] of the methoxy groups to alcoholic groups. The detection of peaks between 2800 and 3000 cm^−1^, corresponding to the methoxy group (–OCH_3_) [88], along with the appearance of O-H peaks in 7′-Methoxy-[1,1′-binaphthalen]-7-ol, suggests that only one of the methyl groups in the molecule was replaced by hydrogen, forming –OH, while the other remained unchanged [72].

### 3.2. Raman Spectroscopy

**Figure 3:**
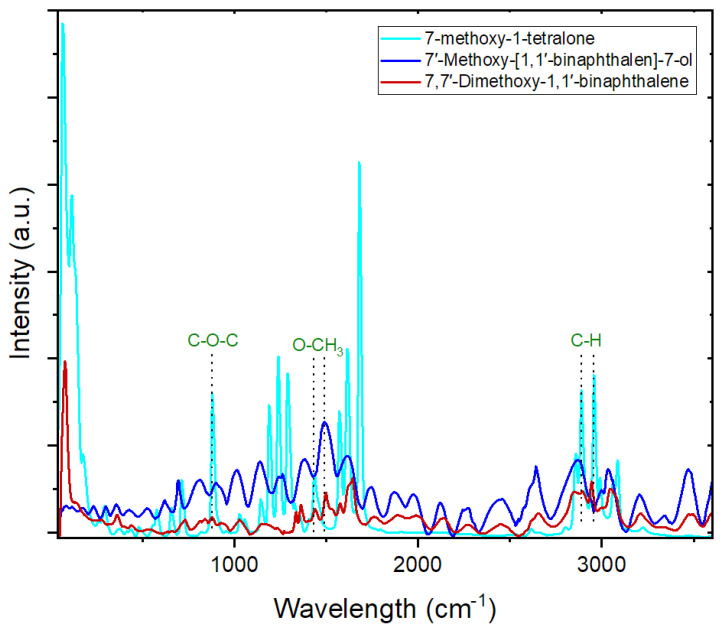
Comparison between the Raman spectra of 7-methoxy-1-tetralone, 7,7′-Dimethoxy-1,1′-binaphthalene, and 7′-Methoxy-[1,1′-binaphthalen]-7-ol.

Raman spectroscopy was employed as another characterisation technique in the comparative analysis of the molecular structures of 7-methoxy-1-tetralone and the developed fluorescence sensor, 7′-Methoxy-[1,1′-binaphthalen]-7-ol (Figure 3). While the spectrum for the initial reagent, 7-methoxy-1-tetralone, was clear with minimum noise, it is imperative to acknowledge the inherent inefficiency of Raman scattering, particularly in the context of fluorescent compounds such as the developed 7′-Methoxy-[1,1′-binaphthalen]-7-ol. In most instances, the generation of a single Raman photon necessitates the incidence of approximately 10^6^–10^8^ laser photons upon the sample [89,90]. Consequently, even minute concentrations of fluorescent species (either in pure compound or as interferences) within the sample can obscure or complicate the detection and interpretation of the weak Raman-scattered photon signals [91,92]. If the fluorescence baseline is elevated, the shot noise emanating from this signal may equate to or even surpass the intensity of the Raman signal itself, effectively veiling the Raman photon signals [91,93].

In an endeavor to rectify this issue, other than minimizing the volume of the sample and changing the scan duration and intensity of the excitation beam [93], we employed the base-line correction of Origin software (v9), employing mathematical techniques aimed at refining the baseline correction [94]. However, it is crucial to underscore that despite these efforts, Raman peaks, specifically the ones associated with 7′-Methoxy-[1,1′-binaphthalen]-7-ol, may still persistently elude detection amidst the backdrop of noise and fluorescence interference.

Looking at Figure 3, the C-O stretching peak for the methoxy group can be seen at approximately 1141 cm^−1^ [95]. The peak at 1239 cm^−1^ could represent C-O symmetric stretching [95]. However, since this frequency is too high, it might correspond to a coupled C-O + O-CH_3_ deformation. The O-CH_3_ bending peaks typically appear between 1450–1475 cm^−1^, but in this case, they are observed at 1432 cm^−1^ for 7-methoxy-1-tetralone and 1487 cm^−1^ for the dimerization products [96]. This shift is likely due to the specific molecular structure of the analysed samples. The CH_3_ symmetric stretching peak is located at 2885 cm^−1^, while the CH_3_ asymmetric stretching peak for the methoxy group is at 2950 cm^−1^ [97,98,99]. The dangling O-H bond of the sensor is centered at 3460 cm^−1^ [100]. The C-O-C symmetric stretching peak is observed at 878 cm^−1^ [95]. The aromatic C-H asymmetric stretching peaks are located at 3079 cm^−1^ for 7-methoxy-1-tetralone and 3042 cm^−1^ for the dimers 7,7′-dimethoxy-1,1′-binaphthalene and 7′-methoxy-[1,1′-binaphthalen]-7-ol [95,96].

### 3.3. Fluorescence Spectroscopy

**Figure 4:**
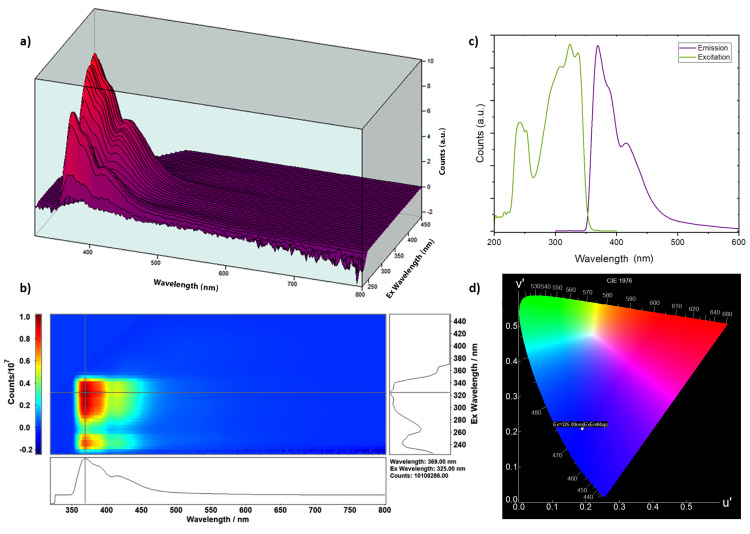
(**a**) The three-dimensional mapping graph illustrates how altering the excitation wavelength affects the intensity of the sensor’s emission peak. (**b**) The contour map displays the fluorescence emission and excitation spectra of the sensor, with the intersection denoting the position of the fluorescence peak (λ_ex_ = 325 nm, λ_em_ = 369 nm). (**c**) A comparison of the emission (λ_ex_ = 325 nm) and excitation (λ_em_ = 369 nm) spectra of 400 ppm 7′-Methoxy-[1,1′-binaphthalen]-7-ol dissolved in methanol. (**d**) The CIE chromaticity plot reveals the color coordinates of the emission beam, demonstrating that the molecule emits blue light upon excitation (λ_ex_ = 325 nm).

Owing to the conjugated double-bond system and the high mobility of their π-electrons, the developed sensor (7′-Methoxy-[1,1′-binaphthalen]-7-ol) was able to exhibit a strong fluorescence emission [64]. The three-dimensional fluorescence spectrum is visually represented in Figure 4a,b, unveiling a prominent fluorescence peak at an emission wavelength of 369 nanometers, which is elicited by an excitation wavelength of 325 nanometers. Subsequently, in Figure 4c, the emission and excitation scans of the sensor at the optimized excitation and emission wavelengths are displayed.

In Figure 4d, the emission peak color resulting from excitation with a 325 nm wavelength is characterized, within the International Commission on Illumination (CIE 1976) color space, as blue. Within this color space, the u’ component corresponds to the u-prime axis, representing hues ranging from green to yellow, while the v’ component signifies the v-prime axis, encompassing hues from blue to red. Collectively, the u’ and v’ components jointly determine the chromaticity coordinates within a cylindrical color space.

**Figure 5:**
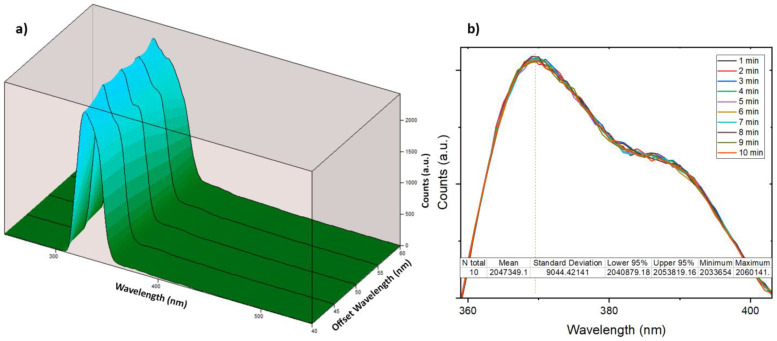
(**a**) The three-dimensional synchronous fluorescence spectroscopy mapping. (**b**) The influence of time on the fluorescence-emission intensity of the developed sensor.

Synchronous fluorescence spectroscopy (SFS) serves as a powerful tool for the detection of impurities or contaminants within chemical samples, even when present at trace levels [101,102,103]. Often, these impurities exhibit distinct fluorescence properties in comparison to the primary product being analyzed. In our study, we utilized SFS to scan the synthesized product, 7′-Methoxy-[1,1′-binaphthalen]-7-ol. Scans encompassed the spectral range from 250 nm to 550 nm, employing 5 nm incremental adjustments within the 40 nm-to-60 nm offset range. The objective was to systematically eliminate the presence of unexpected fluorescence signals, which could serve as indicators of impurities [65,104] (Figure 5a), which suggests the relatively pure product. Simultaneously, to ensure the stability of the developed compound in its solution form over time, and the absence of self-quenching effects [105,106], especially at high concentrations of 7′-Methoxy-[1,1′-binaphthalen]-7-ol, an examination was conducted on the effect of time on the intensity of the emission peak. This involved generating emission spectra spanning the short range (from 350 nm to 420 nm) for a solution containing 500 ppm of the sensor in methanol, recorded over a duration of 10 min (Figure 5b). The resulting data convincingly demonstrate the stability of the sensor’s response, reinforcing its reliability for practical applications.

The morphine molecule features five rings within its three-dimensional structure, with three of these rings lying approximately in the same plane, bonded to hydroxyl groups. Meanwhile, the remaining two rings, including one with oxygen and the other with nitrogen, are oriented at an angle to the others. This configuration, and a nitrogen atom possessing a lone electron pair, provides morphine with the capability to produce fluorescence emissions [64]. To further investigate this property, emission spectra were recorded for standard solutions of pure morphine in methanol. Additionally, the response of the sensor to changes in morphine concentration was observed over a range spanning from 1 ppb to 50 ppm. These observations were subsequently compared with the emission response of the sensor.

**Figure 6:**
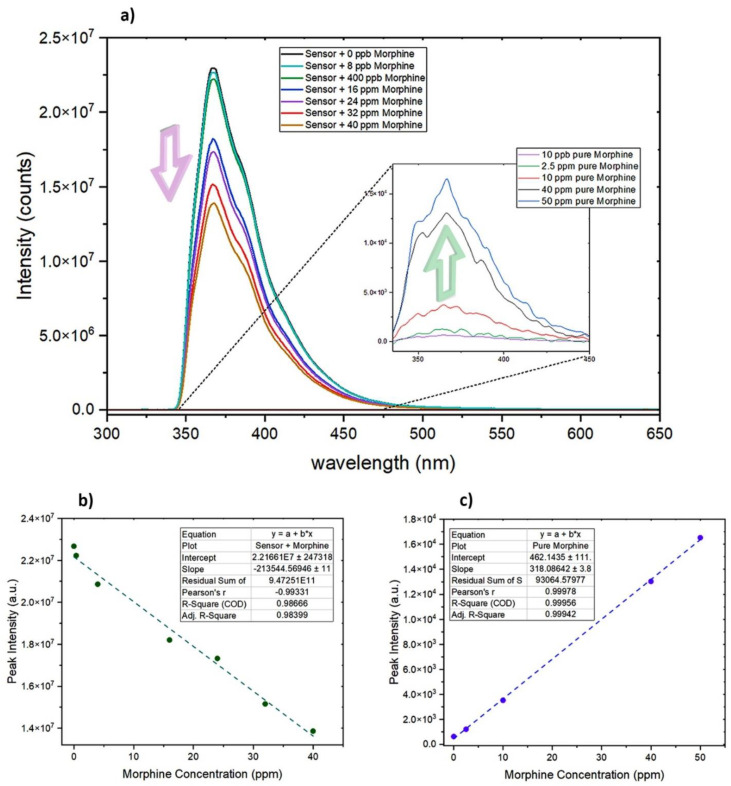
(**a**) Compares the variations in the intensity of the fluorescence-emission peak due to alterations in the concentration of morphine, both in the presence and absence of the developed sensor. (**b**) Illustrates the sensor’s linear range, in response to morphine, displaying an R² value of 0.98 (λ_ex_ = 325 nm). (**c**) Demonstrates the linear range of morphine’s fluorescence peak in the absence of the sensor, accompanied by an R² value of 0.99 (λ_ex_ = 325 nm).

As seen in Figure 6a, pure-morphine standard solutions (in methanol) produced an emission peak of 366 nm (λ_ex_ = 325 nm), which closely matches the fluorescence peak wavelength of 7′-Methoxy-[1,1′-binaphthalen]-7-ol at 369 nm. However, the intensity of the fluorescence emission of the sensor is significantly higher than that of pure morphine. As expected, with an increase in the concentration of morphine in the solution, the intensity of its emission peak shows a linear increase (R^2^ = 0.999; Figure 6c). However, for concentrations lower than 2.5 ppm of the morphine solution (in the absence of sensor), the emission-peak intensity is found to be very low, nearly indistinguishable from noise and the background spectra of pure methanol. At this range, differentiating between noise, the background spectra of pure methanol, and the morphine emission peak becomes challenging.

While both morphine and the developed sensor emit fluorescence peaks at nearly identical wavelengths (λ_morphine_ = 366 nm, λ_sensor_ = 369 nm), the introduction of morphine to the sensor results in quenching, leading to a decrease in the intensity of the emission peak of 7′-Methoxy-[1,1′-binaphthalen]-7-ol. The higher the concentration of morphine, the lower the intensity of the sensor’s emission peak. This intriguing phenomenon has been harnessed to establish a linear relationship between the concentration of morphine and the intensity of the sensor’s fluorescence peak. Notably, the linear range of the turn-off sensor extends from 8 ppb to 40 ppm (R^2^ = 0.987), boasting a remarkable detection limit of 8 ppb. In addition to the observation that 8 ppb was the lowest-tested concentration that caused a noticeable quenching in the sensor response, the detection limit was determined using the formula LoD = 3.3S_b_/m. Here, “S_b_” signifies the standard deviation obtained from three repeated runs (S_b_ = 540.33247), and “m” represents the slope derived from the sensor’s linear graph (m = 213544.57). Consequently, the calculated LoD for the sensor is calculated to be approximately 0.00835 ppm. It is worth highlighting that for concentrations exceeding 40 ppm, the fluorescence peak of pure morphine itself serves as a robust indicator of the presence of a high concentration of morphine in the solution (Figure 6c).

To assess the chemo-selectivity of the developed sensor before examining its behavior in complex matrices such as urine, a series of tests were conducted to observe variations in the sensor’s output signal when exposed to standard solutions containing various drugs. Morphine, ketamine, midazolam, nicotine, and benzyl-piperazine (BZP) share several commonalities despite their distinct effects, uses, mechanisms of action, and therapeutic or recreational purposes [107,108,109,110,111]. Notably, all of them feature structures composed of multiple aromatic rings and harbor active functional groups, allowing these substances to primarily exert their influence on the central nervous system. They cause the modulation of brain function and neurotransmitter activity, resulting in alterations in perception, mood, consciousness, or pain perception, and all of them have the potential for abuse, which can lead to addiction or dependence [7].

**Figure 7:**
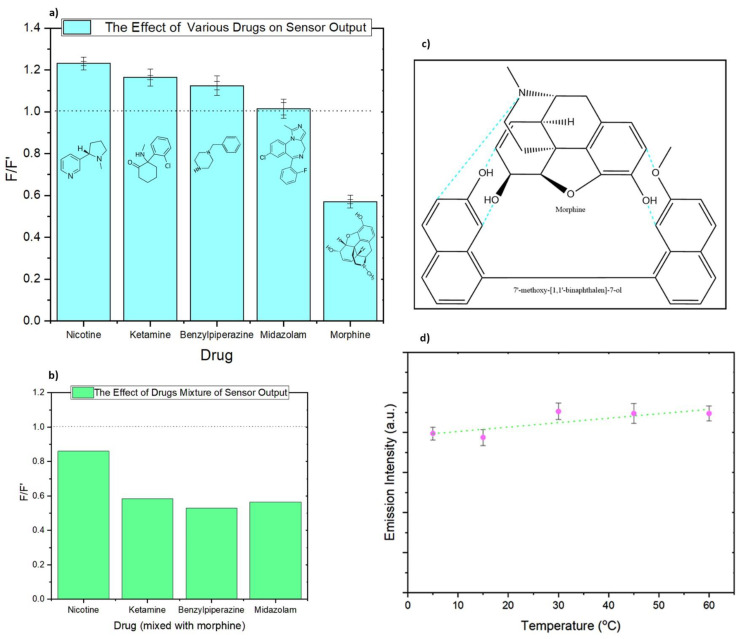
(**a**) Bar chart illustrating variations in sensor fluorescence when exposed to various drugs (20 ppm each) individually. F represents the fluorescence response of the sensor in the presence of the drug, while F’ signifies the sensor’s fluorescence response in the absence of any drugs. (**b**) The competition experiment on the effect of other drugs on morphine detection by developing a mixture of morphine (20 ppm) with other drugs (20 ppm each) separately. (**c**) The possible reaction sites for the formation of a non-fluorescent complex between morphine and the sensor. (**d**) Effect of temperature on fluorescence-emission intensity of the sensor in the presence of a constant amount of morphine (20 ppm in methanol).

Standard solutions with a concentration of 20 ppm for each of these drugs were individually prepared in methanol. The sensor’s response in the absence and presence of these drugs was utilized to demonstrate the selectivity of the sensor, as depicted in Figure 7a. Interestingly, only morphine induced quenching effects, while the other tested drugs either did not alter the fluorescence-emission intensity of the sensor or caused an increase in emission intensity. The observed increase in intensity, as seen with molecules such as ketamine and nicotine, could be attributed to changes in the local environment surrounding the sensor molecule or the stabilization resulting from the formation of more complex structures. In another experiment aimed at assessing the sensor’s affinity for morphine, standard solutions were prepared by combining previously tested drugs (each at 20 ppm) with morphine (20 ppm). Subsequently, the sensor’s response to morphine within this mixture was evaluated. Figure 7b presents the results, indicating a notable affinity of the sensor for morphine compared to all other tested drugs. While fluorescence quenching of the sensor is observed in all mixed samples upon the presence of morphine (F/F′), it is also noticeable by the comparison between Figure 7a,b. The fluorescence intensity in the nicotine mixture sample, despite showing quenching, is comparatively higher than in other mixed samples. This observation may suggest a potential competition between nicotine and morphine molecules for binding sites on the developed sensor.

When two different fluorescent molecules, both serving as sensor and analyte, are brought into close proximity, they can exhibit a phenomenon in which one molecule quenches the fluorescence of the other. This quenching can occur through various mechanisms, primarily involving energy-transfer processes. One of the most prevalent mechanisms responsible for this phenomenon are Förster Resonance Energy Transfer (FRET) [64]. Since the emission wavelength of morphine (366 nm) is significantly distant (more than 10 nm apart) from the excitation wavelength of the sensor (325 nm) and does not exhibit spectral overlap with it, it precludes the possibility of an energy transfer from one fluorescent molecule (the donor) to another (the acceptor) [112]. This energy-transfer mechanism, relies on spectral overlap for effective quenching of the donor molecule’s fluorescence and is thus ruled out in this context.

Dynamic quenching, collisional quenching, and photo-induced electron transfers (PETs) are temperature-dependent processes [64]. Typically, PET rates increase with higher temperatures due to enhanced molecular motion and collision rates. However, it is important to note that no significant changes in the emission-peak intensity of the sensor, while maintaining a constant concentration of morphine, are observed in response to variations in temperature (Figure 7d). This indicates that the quenching has occurred due to a strong binding interaction between the sensor and quencher (morphine). It can result from the formation of a non-fluorescent complex through non-covalent interactions, such as van der Waals forces, hydrogen bonding, or electrostatic interactions, between 7′-Methoxy-[1,1′-binaphthalen]-7-ol and morphine molecules (Figure 7c) [64,113].

Morphine (Figure 7a,c) exhibits the potential to engage in various non-covalent interactions, contributing to its ability to induce static quenching with the sensor. These interactions include the formation of van der Waals bonds facilitated by its aromatic carbon rings (rings comprising carbons 1, 2, 7, and 8), the establishment of hydrogen bonds using its alcohol groups (located on carbons 3 and 6), and the possibility of electro-static interactions, primarily through its tertiary nitrogen atom, which is connected between Carbons 9 and 16. This multifaceted nature of morphine’s molecular structure, which makes it interact with μ opioid receptors in the body [114], makes it a particularly suitable candidate for causing static quenching phenomena in this context.

In addition, 7′-Methoxy-[1,1′-binaphthalen]-7-ol (Figure 1) comprises four aromatic rings in its structure, endowing it with the capability to engage in van der Waals interactions. Furthermore, its planar configuration enables the sensor to establish stacking interactions with other planar or nearly planar molecules. Additionally, the presence of oxygen atoms within the molecule, found in both the alcohol and methoxy moieties, facilitates the formation of potent non-covalent bonds, namely hydrogen bonds, with other molecules. The sensor’s remarkable selectivity for morphine, as demonstrated in Figure 7a, when compared to four other tested drugs (benzylpiperazine, ketamine, midazolam, and nicotine), strongly implies that multiple types of non-covalent bonds may be contributing to the static quenching of the sensor.

### 3.4. Comprehensive Urine Analysis

The current standard procedure for opiate urine testing involves the collection of urine samples, their transfer to laboratories, and a subsequent analysis using liquid chromatography with tandem mass spectrometry (LC-MS/MS) [115,116,117]. Detecting the presence of morphine, whether as the primary drug of use or as a metabolite of other substances like heroin, in urine becomes exceedingly challenging after 48 h due to its limited half-life of 2–4 h. This underscores the critical importance of analyzing collected samples and obtaining results as swiftly as possible. Therefore, any techniques capable of reducing the sample transportation time from the collection site to the laboratory or delivering faster results compared to conventional methods like LC-MS/MS would be highly advantageous.

**Figure 8:**
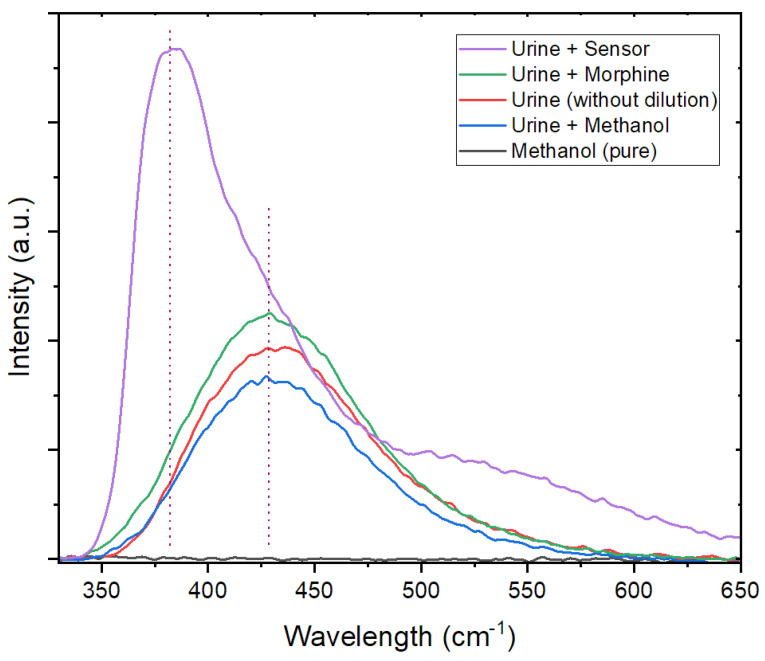
Comparison of fluorescence-emission spectra. The graph illustrates the fluorescence-emission spectra of the sensor (100 ppm in methanol) in a 1:1 mixture with urine, morphine (40 ppm in methanol) in a 1:1 mixture with urine, pure methanol in a 1:1 mixture with urine, pure urine without any additives, and pure analytical-grade methanol.

As demonstrated earlier, our objective was to develop a selective molecular fluorescence sensor suitable for use with both portable spectrofluorometers, ensuring accurate on-site testing, and stationary spectrofluorometers, providing faster responses than techniques such as LC-MS/MS. To verify that the sensor can still produce a distinguishable emission spectrum, unobstructed by the emissions of other molecules within the complex urine matrix, a series of tests were conducted, as depicted in Figure 8.

The results indicate a significant difference in peak wavelength between pure urine (428 nm) and the emission from the sensor (382 nm) within the urine matrix. The small shift in the fluorescence emission of the sensor (13 nm blue shift) can be attributed to alterations in the solvent’s polarity, viscosity, and hydrogen-bonding characteristics [64,118]. Notably, methanol dilution does not diminish the fluorescence intensity of the urine. However, the introduction of a morphine standard solution (in methanol) to the urine sample leads to an increase in the fluorescence intensity of the broad urine-emission peak.

**Figure 9:**
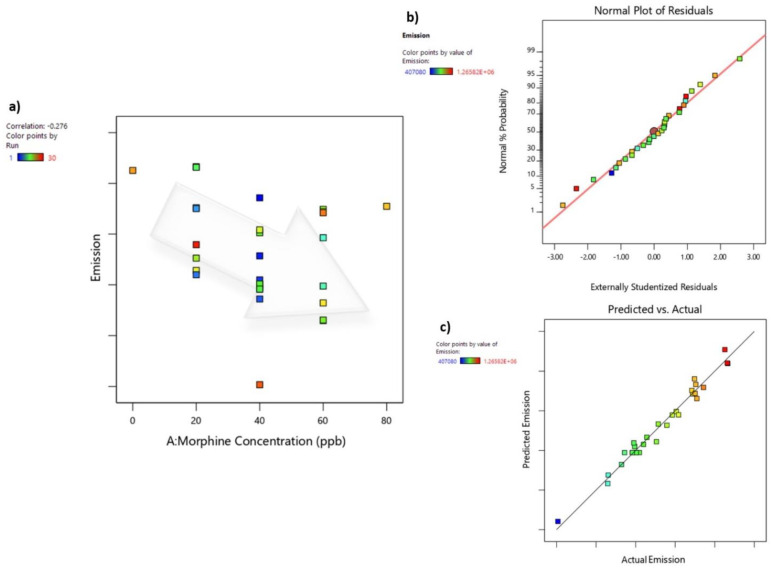
(**a**) Sensor’s response to morphine concentration: This panel illustrates the dynamic response of the sensor as it detects variations in morphine concentration within urine samples. (**b**) Normal plot of residuals: In this plot, the residuals are visually examined for adherence to normal distribution assumptions, providing insights into the model’s performance. (**c**) Predicted-versus-actual graph: This graph showcases the predictive accuracy of the model by comparing the predicted sensor’s fluorescence emission with the actual values.

While the increase in the concentration of morphine in urine samples still leads to a linear reduction in the sensor’s fluorescence emission (Figure 9a), the chemometric technique of central composite design-response surface methodology was utilized to examine the influence of various variables on the sensor’s response within the complex urine sample. The aim was to identify and optimize key operational parameters to enhance sensor performance. It is worth mentioning that due to the small peak shift resulting from changes in the solvent, as discussed earlier, the alterations in the intensity of the emission peak at 382 nm were used for CCD-RSM analysis.

In Table 2, the design matrix and outcomes were presented, stemming from the central composite full-factorial experimental design. The primary focus of this investigation was centered on the assessment of reproducibility and errors, specifically at the “center points” of the design. The “factorial points”, constituting 2^k^ data points (with k representing the involvement of four factors), were strategically employed to facilitate the estimation of first-order effects and two-factor interactions. Additionally, the “axial points”, comprising 2k data points, were utilized to gauge the exclusive quadratic effects within the experimental framework. Leverage quantifies the potential for a data point to exert a strong influence on the regression analysis, and it depends on the predictor values; high leverage points (close to 1.0) are considered outliers with respect to the independent variables. The average leverage of the 30 runs was 5.0 × 10^−1^, and neither leverage was 1.0 or higher.

The optimization of the sensor efficiency was achieved through the application of response-surface methodology. Within this framework, we employed an analysis of variance (ANOVA) in the context of a standard response surface design to investigate the key parameters affecting the performance of the electrochemical sensor. In this study, we opted for a quadratic model due to its capacity to accommodate higher-order polynomial terms that carry significant implications, while the cubic model was rendered inconclusive. The results of the ANOVA, presented in Table 3, offer valuable insights into the significance of each variable. The Model F-value, which stands at 44.86, underscores the overall significance of our model. The probability of encountering such a substantial F-value purely by chance is a mere 0.01%. *p*-values below 0.0500 indicate that the model terms hold substantial importance. In our investigation, the terms A, C, D, BC, A², B², and D² were all found to be statistically significant. Conversely, values exceeding 0.1000 signify that the model terms are not statistically significant. In cases where numerous terms fall into this category, excluding those essential for maintaining the hierarchy, may enhance model performance [119,120,121]. To assess the suitability of the model, we conducted a Lack of Fit analysis, which should ideally yield an insignificant result for the model to align effectively with the experimental design. In our study, the Lack of Fit F-value, which is 3.65, does not reach statistical significance, affirming the adequacy of our model.

Upon analyzing the data, it becomes evident that the Predicted R², standing at 0.8778, aligns reasonably well with the Adjusted R², which reaches 0.9549. This implies that the difference between the two values is less than 0.2, signifying a close match. Additionally, the Adequacy Precision analysis, which gauges the signal-to-noise ratio, reveals that our model boasts a ratio of 28.462, which is well above the desirable threshold of 4. This strong signal-to-noise ratio reaffirms the model’s suitability for navigating the design space.

Furthermore, the equation expressed in terms of coded factors serves as a valuable tool for predicting responses based on various factor levels. This coded equation allows for a comparative assessment of the relative impact of individual factors through the examination of their coefficients. The equation is as follows:

$$\begin{array}{ll} Emission= & 789,026.99-\left(61,501.38\times A\right)-\left(15,784.00\&\times B\right)+\left(169,072.98\times C\right)-\left(35,336.13\times D\right)\\ & +\left(7404.21\times AB\right)+\left(14,880.30\times AC\right)-\left(15,238.75\times AD\right)-\left(41,704.39\times \&BC\right)\\ & -\left(11,917.54\times \&BD\right)+\left(9447.28\times \&CD\right)+\left(99,043.50\times A^{2}\right)+\left(43,927.54\&\times B^{2}\right)\\ & -\left(2254.98\&\times C^{2}\right)+(29,954.79\times D^{2}) \end{array}$$

In essence, these findings underscore the robustness of our model, making it a reliable tool for navigating the intricacies of the design space and providing valuable insights into the impact of individual factors on emissions.

The normal plot of residuals serves as a valuable tool for assessing the assumption of normality in error-term distribution [122,123]. This plot employs a diagonal line, aligning with the lower and upper quartiles of the theoretical distribution, to visually gauge the linearity of the relationship between theoretical and sample percentiles. When this relationship appears approximately linear, as suggested by the outcome plot of this study (Figure 9b), it indicates that the error terms follow a normal distribution. Consequently, we can confidently proceed with the assumption that the error terms exhibit normal distribution properties [120,121,124]. Another essential visualization tool is the actual-versus-predicted emission graph (Figure 9c), which offers a visual assessment of the model’s fit by depicting variations attributed to random effects [125,126]. This graph plots observed emission values against the predicted emission values. In an ideal scenario, data points would be evenly distributed along the 45-degree line. A well-fitted model would exhibit data points closely aligned with the fitted line. It is worth noting that the residual, defined as the difference between actual and predicted (or fitted) response variable values, should not be confused with the normal residual plot in this context. Both plots, as depicted in Figure 9b,c, confirm that there are no anomalies in the experimental data, and they align well with the predicted data within the quadratic model. This robust alignment proves the model’s effectiveness in accurately predicting fluorescence quenching.

**Figure 10:**
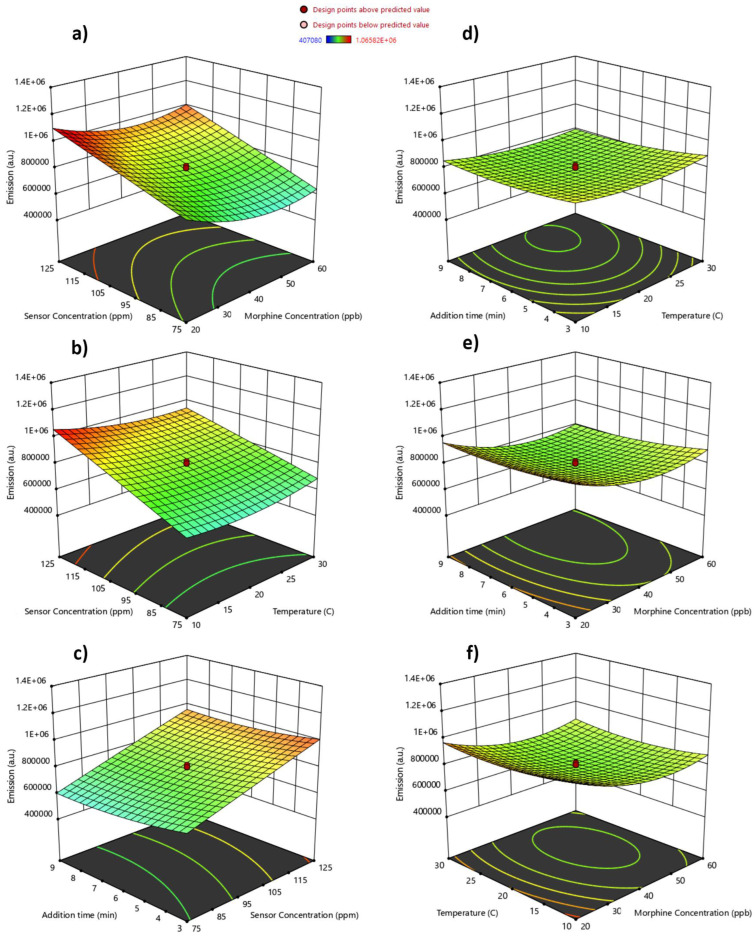
Three-dimensional plots of sensor’s fluorescence-emission response in urine samples: (**a**) interactive effect of morphine’s concentration and temperature; (**b**) interactive time and morphine’s concentration; (**c**) interactive effect of time and temperature; (**d**) interactive effect of sensor’s concentration and morphine’s concentration; (**e**) interactive effect of sensor’s concentration and temperature; (**f**) interactive effect of time and sensor’s concentration.

To gain a deeper understanding of the variables with the most significant influence on quenching, ultimately resulting in minimized emissions, and to explore the interactions between these variables, we turn our attention to Figure 10a–f. These graphical representations provide a valuable tool for predicting optimal conditions, and the presence of curvatures in these response surfaces hints at intricate interactions among the variables.

As expected, Figure 10a vividly illustrates a trade-off relationship between the concentration of morphine and the sensor’s concentration. As the morphine concentration increases, there is a notable reduction in emissions, while elevating the sensor’s concentration amplifies fluorescence emission at the specified peak wavelength. This behavior aligns seamlessly with our initial expectations, and a similar trend can be observed in Figure 10b,c. Remarkably, the enhancement of the sensor’s concentration emerges as a pivotal factor influencing fluorescence intensity, as anticipated. However, it is worth noting that changes in morphine concentration have a significant impact even within ppb ranges, whereas sensor-concentration changes operate at ppm levels. Conversely, two variables, mixing time and temperature (Figure 10d), exhibit a relatively weak mutual influence, yielding a nearly flat response with only a slight increase in quenching observed between temperatures of 20–25 °C, coupled with a modest extension in mixing time. However, when examining the interplay between mixing time and morphine concentration on emissions (Figure 10e), a somewhat distinct relationship emerges. For higher morphine concentrations, a slightly prolonged mixing time before initiating fluorescence studies appears to enhance quenching effects. Additionally, Figure 10f demonstrates that the sensor exhibits substantial quenching as the morphine concentration increases, particularly within the temperature range of approximately 15–30 °C. These findings provide valuable insights into the intricate interactions of these variables and their impact on the fluorescence emissions of the sensor.

Understanding the intricate interplay among these variables and their impacts on the sensor’s emissions is invaluable. Consequently, we can deduce that the primary influencers in the realm of fluorescence quenching and emission in urine are the concentrations of morphine and the sensor, with nuanced interaction effects discernible between them. Mixing time, in this context, assumes a subsidiary role, while temperature exerts the least amount of influence on these processes. Armed with this knowledge, we can tailor the optimization of sensors for urine matrices, meticulously considering all variables and the magnitude of their effects.

Building on this comprehension, we delineate our desired conditions with precision. Our primary objective is to achieve the lowest possible emission, indicative of the highest quenching effect, while concurrently minimizing the concentration of morphine. In this pursuit, we must also consider the economic aspects, encompassing the affordability and production costs of the sensor. Consequently, one of our paramount desirability settings involves determining the minimum requisite amount of the sensor. Our desirability scale ranges from 0.0 (indicating undesirability) to 1.0 (representing high desirability), and this configuration allows us to reach an optimal condition with a commendable desirability rate of 0.825.

**Figure 11:**
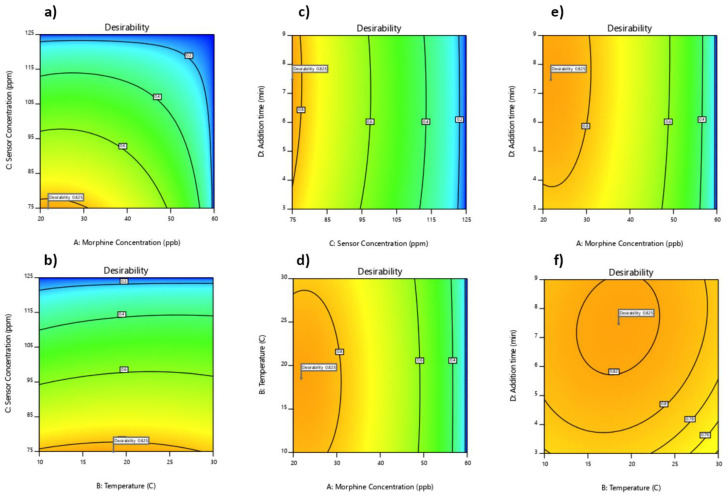
Two-dimensional desirability graphs versus actual points: (**a**) Actual factors include temperature at 18.53 °C and addition time at 7.47 min; (**b**) Actual factors include morphine’s concentration at 21.83 ppb and addition time at 7.47; (**c**) Actual factors include morphine’s concentration at 21.83 ppb and temperature at 18.53 °C (**d**) Actual factors include sensor’s concentration at 75 ppm and addition time at 7.47 min; (**e**) Actual factors include temperature at 18.53 °C and sensor’s concentration at 75 ppm; (**f**) Actual factors include morphine’s concentration at 21.83 ppb and sensor’s concentration at 75 ppm.

As depicted in Figure 11a–f, it becomes evident that the lowest concentration of morphine capable of eliciting maximum quenching in a urine matrix is precisely 21.81 ppb. Moreover, the optimal working conditions include a temperature of 18.48 degrees Celsius and a waiting time (after mixing) of 7.45 min, both of which yield the smallest quenching effect.

To validate the outcomes derived from the modeled response and to ensure their reliability, a confirmation sample was employed. A confirmation sample comprises a series of supplementary trials conducted at a specific combination of factor settings, situated within the studied range and aligned with the optimized conditions. The average response obtained from this confirmation sample is then compared to the prediction interval (PI). When the average observation from the confirmation experiment falls within the prediction interval, it affirms the accuracy and dependability of the model.

For this purpose, three separate samples were prepared and analyzed using a spectrofluorometer. The selected parameter settings were as follows: a morphine concentration of 21.8 ppb, a sensor concentration of 75 ppm, a temperature of 18.5 °C, and an additional mixing time of 7.5 min. The resulting data are summarized in Table 4, where a 95% confidence level was applied. It is noteworthy that all measured responses, as well as the mean of repeated tests, fall comfortably within the prediction intervals of the quadratic model. This comprehensive alignment serves to confirm the precision of the quadratic model and the replicability of the sensor’s results.

### 3.5. Evaluating Morphine Sensors in Comparison

As previously discussed, morphine holds a critical role as a potent analgesic medication, particularly in medical contexts like surgical procedures [127]. For effective pain relief, it is recommended that the optimal dose should not exceed 15 mg per 70 kg of body weight when administered through an extended-release liposome injection or an intrathecal injection [128,129,130,131,132]. In the case of oral administration, the maximum daily dose can reach as high as 30 mg, considering the effects of first-pass metabolism [133]. It is worth noting that there is not a well-defined toxic dose or specific plasma/blood concentration for morphine [44]. However, its use carries a substantial risk of addiction and misuse, contributing to a range of serious health complications [134]. Importantly, the administration of higher doses of morphine can lead to respiratory depression and potentially life-threatening health issues [135]. The use of morphine for analgesia has been associated with a significant number of drug-related deaths, with estimated incidence rates ranging from 0.3% to 4% [44,136]. Additionally, morphine can be detected in trace amounts not only in biological fluids but also in aqueous environments [137]. Consequently, considerable research efforts have been dedicated to the development of innovative detection techniques and sensors to rapidly and accurately quantify morphine in various sample types. In this context, we seek to compare the sensor developed in our research with 15 other different sensing systems, as presented in Table 5. This comparison aims to highlight the distinct advantages of our turn-off sensor in terms of its performance and capabilities.

Upon comparing the sensors detailed in Table 5, it becomes evident that our developed sensor boasts several distinct advantages. With a detection limit as low as 8 ppb, our fluorescence sensor not only rivals but also surpasses established methods such as GC-MS, which has a detection limit of 3 ppb [138]. This is particularly noteworthy due to the inherent ease of use and affordability associated with fluorescence spectroscopes. In addition to its impressive sensitivity, our sensor offers the distinct advantage of providing rapid results in under 10 s. Furthermore, the biological (urine) sample-preparation process is remarkably straightforward and swift, involving the addition of the sensor solution to urine and a brief 7.45-min mixing period before measuring emissions (as depicted in Figure 11c,e,f) to achieve highly optimized outcomes. This results in a total analysis time of less than 7.5 min. Moreover, our sensor exhibits a significantly wider linear range, surpassing the capabilities of all other sensors in the comparison.

The developed sensor boasts an impressive linear range that spans from 0.008 to 40 ppm, a range that is more extensive than nearly all other methods considered, including GC-MS (0.0025–2 ppm) [138], surface plasmon resonance imaging (1–50 ppm) [142], colorimetric assay (19.97–856 ppb) [143], piezoelectric sensor (0.25–2500 ppb) [140], and various other sensor types [62,139,141,146]. This practical broader linear range enhances the versatility and adaptability of our sensor for use with a wide range of concentration levels.

Notably, the detection limit, linear range, and response time of our fluorescence sensor are on par with electrochemical morphine sensors [26,144,145], even though they employ a different detection method. It is worth mentioning that the significantly wider linear range offered by our sensor in this study surpasses that of all other morphine fluorescence sensors [56,147,148], including the fluorescein-gold nanoparticle-based fluorescence sensor, which, despite its competitive detection limits (0.015 ppb), suffers from a limited linear range (0.001–13.9 ppb) [149].

## 4. Conclusions

This study embarked on the comprehensive journey of synthesizing and characterizing 7′-Methoxy-[1,1′-binaphthalen]-7-ol from scratch, subjecting it to rigorous characterisation and purity tests employing spectroscopy techniques. During this investigation, we unveiled its remarkable photoluminescence properties, notably the ability to emit intense, blue-colored fluorescence. This emission peak became the focal point of our research, as we explored its potential as a fluorescence sensor for morphine. Our findings revealed that it exhibited quenching exclusively in the presence of morphine without responding to other tested drugs or a range of diverse molecules typically found in complex samples like urine.

After confirming these initial results, we harnessed CCD-RSM to not only delve into the intricate interplay of various variables and their influence on each other but also to optimize the testing conditions for the sensor. Our objective was to achieve maximum quenching with the lowest possible concentration of morphine. Subsequently, we rigorously assessed the reliability and reproducibility of the developed quadratic model.

In the subsequent comparative analysis with a range of other morphine sensors, our sensor was found to excel in terms of response time, ease of use, linear range, and detection limit. This promising performance positions it as a compelling choice for morphine quantification in various sample types, potentially surpassing more complex and costly analytical methods. Due to these achievements, our developed sensor holds significant promise as an affordable, user-friendly, and reliable detection technique for morphine concentration measurements in biological fluids, particularly urine. Its potential applications span forensic and medical studies where it can serve as a valuable tool for precise morphine quantification.

## Figures and Tables

**Table 1:** Experimental factors and levels in the central composition design.

Notation	Factor	Unit	Range and Levels				
			−α	−1	0	+1	+α
A	Concentration of morphine in the solution	ppb	0	20	40	60	80
B	Temperature of the solution	°C	0	10	20	30	40
C	Concentration of the sensor in the solution	ppm	50	75	100	125	150
D	Waiting time after addition of morphine	min	0	3	6	9	12

**Table 2:** Design matrix and the results of the central composite full factorial design.

Run	A	B	C	D	Leverage	Response (Emission)	Space Type
1	40	20	150	6	5.833 × 10^−1^	1.14 × 10^6^	Factorial
2	40	40	100	6	5.833 × 10^−1^	1.01 × 10^6^	Factorial
3	40	20	100	6	1.667 × 10^−1^	8.19 × 10^5^	Factorial
4	40	20	100	6	1.667 × 10^−1^	1.667 × 10^−1^	Factorial
5	20	10	75	9	5.833 × 10^−1^	8.40 × 10^5^	Factorial
6	20	30	125	9	5.833 × 10^−1^	1.26 × 10^6^	Factorial
7	40	20	100	6	1.667 × 10^−1^	7.83 × 10^5^	Factorial
8	20	30	125	3	5.833 × 10^−1^	1.27 × 10^6^	Center
9	60	10	75	9	5.833 × 10^−1^	6.59 × 10^5^	Factorial
10	60	30	125	9	5.833 × 10^−1^	1.10 × 10^6^	Factorial
11	60	30	75	3	5.833 × 10^−1^	7.29 × 10^5^	Factorial
12	40	20	100	12	5.833 × 10^−1^	7.91 × 10^5^	Factorial
13	40	20	100	6	1.667 × 10^−1^	8.01 × 10^5^	Center
14	20	10	125	9	5.833 × 10^−1^	1.10 × 10^6^	Center
15	40	20	100	6	1.667 × 10^−1^	7.83 × 10^5^	Center
16	40	20	100	6	1.667 × 10^−1^	8.04 × 10^5^	Factorial
17	40	0	100	6	5.833 × 10^−1^	1.02 × 10^6^	Factorial
18	60	30	75	9	5.833 × 10^−1^	7.95 × 10^5^	Factorial
19	60	10	125	3	5.833 × 10^−1^	1.08 × 10^6^	Factorial
20	20	30	75	9	5.833 × 10^−1^	8.57 × 10^5^	Factorial
21	60	30	125	3	5.833 × 10^−1^	9.86 × 10^5^	Axial
22	40	20	100	0	5.833 × 10^−1^	9.14 × 10^5^	Center
23	20	10	75	3	5.833 × 10^−1^	9.05 × 10^5^	Axial
24	60	10	75	3	5.833 × 10^−1^	6.61 × 10^5^	Axial
25	80	20	100	6	5.833 × 10^−1^	1.11 × 10^6^	Axial
26	0	20	100	6	5.833 × 10^−1^	1.25 × 10^6^	Axial
27	60	10	125	9	5.833 × 10^−1^	1.09 × 10^6^	Axial
28	40	20	50	6	5.833 × 10^−1^	4.07 × 10^5^	Center
29	20	10	125	3	5.833 × 10^−1^	1.10 × 10^6^	Axial
30	20	30	75	3	5.833 × 10^−1^	9.58 × 10^5^	Axial

**Table 3:** The ANOVA results of CCD for the developed morphine sensor in urine samples.

Source	Sum of Squares	Degree of Freedom	Mean Square	F-Value	*p*-Value	
**Model**	1.16 × 10^12^	14	8.31 × 10^10^	44.9	1.16 × 10^−9^	*significant*
A-Morphine’s Concentration	9.08 × 10^10^	1	9.08 × 10^10^	49	4.29 × 10^−6^	
B-Temperature	5.98 × 10^9^	1	5.98 × 10^9^	3.23	9.26 × 10^−2^	
C-Sensor’s Concentration	6.86 × 10^11^	1	6.86 × 10^11^	370	5.52 × 10^−12^	
D-Addition time	2.99 × 10^10^	1	2.99 × 10^10^	16.2	1.11 × 10^−3^	
AB	8.77 × 10^8^	1	8.77 × 10^8^	0.473	5.02 × 10^−1^	
AC	3.54 × 10^9^	1	3.54 × 10^9^	1.91	1.87 × 10^−1^	
AD	3.72 × 10^9^	1	3.72 × 10^9^	2.01	1.77 × 10^−1^	
BC	2.78 × 10^10^	1	2.78 × 10^10^	15	1.50 × 10^−3^	
BD	2.27 × 10^9^	1	2.27 × 10^9^	1.23	2.86 × 10^−1^	
CD	1.43 × 10^9^	1	1.43 × 10^9^	0.771	3.94 × 10^−1^	
A²	2.69 × 10^11^	1	2.69 × 10^11^	145	4.08 × 10^−9^	
B²	5.29 × 10^10^	1	5.29 × 10^10^	28.6	8.19 × 10^−5^	
C²	1.39 × 10^8^	1	1.39 × 10^8^	0.0753	7.88 × 10^−1^	
D²	2.46 × 10^10^	1	2.46 × 10^10^	13.3	2.40 × 10^−3^	
**Residual**	2.78 × 10^10^	15	1.85 × 10^9^			
Lack of Fit	2.44 × 10^10^	10	2.44 × 10^9^	3.65	8.2753 × 10^−2^	*not significant*
Pure Error	3.35 × 10^9^	5	6.69 × 10^8^			
Cor Total	1.19 × 10^12^	29				

**Table 4:** Quadratic model’s confirmation data.

Response Type	Predicted Mean	Predicted Median	Number of Runs	SE Pred ^a^	95% PI Low	Data Mean	95% PI High
Emission	7.59987 × 10^5^	7.59987 × 10^5^	3	3.25448 × 10^4^	6.90620 × 10^5^	8.11434 × 10^5^	8.29355 × 10^5^

^a^ Standard deviation associated with the prediction of observations.

**Table 5:** Comparison table of different morphine-sensing technologies.

Detection Method	Sensing Material	Detection Limit	Linear Range	Analysis Duration ^a^	Complex Sample	Ref.
Gas chromatography–mass spectrometry	*Not available*	3 ppb	0.0025–2 ppm	<70 min	Urine	[138]
SPME ^b^ RP-HPLC ^c^ and LC-MS/MS	Carboxylated carbon nanotubes	1 ppb	1–10 ppb and 0.001–1 ppm ^d^	30 min	Ferula gummosa ^e^	[139]
Piezoelectric biosensor	Anti-Morphine antibody on the gold coated quartz	0.25 ppb	0.25–2500 ppb	8 min	Urine	[140]
Magnetic resistance sensory	Superparamagnetic nanoparticles	0.1 ppb	0.5–1.5 ppb	10 s	*None*	[141]
Surface plasmon resonance imaging	Activated carboxyl groups on the chips	9.59 ppb	1–50 ppm	20 min	Urine	[142]
Colorimetric determination	Melamine modified gold nanoparticles	4.85 ppb	19.97–856 ppb	10 min	Urine and serum	[143]
Colorimetric determination	Au@Ag core–shell nanoparticles	55 ppb	0.055–30 ppm	5 min	Urine	[62]
Square wave voltammetry	Mesoporous carbon nanostructures	7.7 ppb	0.0285–114.136 ppm	>2 min	Urine	[144]
Differential pulse voltammetry	Gold nanodendrites—broken hollow carbon spheres	2.37 ppb	0.0029–85.6 ppm	<10 s	Human serum and saliva	[145]
Differential pulse voltammetry	Hierarchical CoO_4_-carbon composite	25.68 ppb	0.228–21.4 ppm	6 min	Urine and serum	[146]
Linear sweep voltammetry	Highly boron-doped BCN (*p*-BCN)	5.08 ppb	0.014–57.07 ppm	<10 s ^f^	Human serum	[26]
Up-conversion luminescent system	Nitrocellulose membrane	0.1 ppb	0.1–10 ppb	>30 s	Human hair	[147]
Capillary zone electrophoresis with fluorescence detection	Disodium tetraborate decahydrate (BGE solution)	0.5 ppb	0.002–2 ppm	>32 min	Urine	[148]
Ratiometric fluorescence sensor	Nitrogen-doped carbon dot	71.8 ppb	0.25–25 ppm	<31 min	Human plasma	[56]
Turn-on fluorescence detection	Fluorescein—Gold nanoparticles	0.015 ppb	0.0013–13.942 ppb	6 min	Urine and serum	[149]
**Fluorescence quenching (turn-off) system**	**7′-Methoxy-[1,1′-binaphthalen]-7-ol**	**8 ppb**	**0.008–40 ppm**	**<10 s ^g^**	**Urine**	**This work**

^a^ Including pretreatment time for complex biological samples. ^b^ Solid-phase microextraction. ^c^ Reversed-phase high-performance liquid chromatography. ^d^ Two different linear ranges. ^e^ Dried herb. ^f^ The electrolyte solution had its dissolved oxygen removed by introducing nitrogen gas into the buffer solution for a duration of 20 min prior to each experiment. ^g^ Sensor can generate an acceptable and measurable response in less than 10 s, but allowing for 7.4 min mixing time with urine will offer the most optimized response.

## Data Availability

Data are contained within the article.
